# Exploring Jahn-Teller distortions: a local vibrational mode perspective

**DOI:** 10.1007/s00894-024-05882-8

**Published:** 2024-03-13

**Authors:** Mateus Quintano, Renaldo T. Moura, Elfi Kraka

**Affiliations:** 1 Computational and Theoretical Chemistry Group (CATCO), Department of Chemistry, Southern Methodist University, 3215 Daniel Ave, Dallas, TX 75275-0314 USA; 2 Department of Chemistry and Physics, Center of Agrarian Sciences, Federal University of Paraiba, Areia, PB 58397-000 Brazil

**Keywords:** Jahn-Teller effect, Vibrational spectroscopy, Local vibrational mode theory, CNM/ACS protocol

## Abstract

**Abstract:**

The characterization of normal mode (CNM) procedure coupled with an adiabatic connection scheme (ACS) between local and normal vibrational modes, both being a part of the Local Vibrational Mode theory developed in our group, can identify spectral changes as structural fingerprints that monitor symmetry alterations, such as those caused by Jahn-Teller (JT) distortions. Employing the PBE0/Def2-TZVP level of theory, we investigated in this proof-of-concept study the hexaaquachromium cation case, $[\text {Cr}{(\text {OH}_{2})}_{6}]^{3+}$/$[\text {Cr}(\text {OH}_{2})_{6}]^{2+}$, as a commonly known example for a JT distortion, followed by the more difficult ferrous and ferric hexacyanide anion case, $[\text {Fe}{(\text {CN})}_{6}]^{4-}$/$[\text {Fe}{(\text {CN})}_{6}]^{3-}$. We found that in both cases CNM of the characteristic normal vibrational modes reflects delocalization consistent with high symmetry and ACS confirms symmetry breaking, as evidenced by the separation of axial and equatorial group frequencies. As underlined by the Cremer-Kraka criterion for covalent bonding, from $[\text {Cr}{(\text {OH}_{2})}_{6}]^{3+}$ to $[\text {Cr}{(\text {OH}_{2})}_{6}]^{2+}$ there is an increase in axial covalency whereas the equatorial bonds shift toward electrostatic character. From $[\text {Fe}{(\text {CN})}_{6}]^{4-}$ to $[\text {Fe}{(\text {CN})}_{6}]^{3-}$ we observed an increase in covalency without altering the bond nature. Distinct $\pi$ back-donation disparity could be confirmed by comparison with the isolated $\text {CN}^{-}$ system. In summary, our study positions the CNM/ACS protocol as a robust tool for investigating less-explored JT distortions, paving the way for future applications.

**Graphical abstract:**

The adiabatic connection scheme relates local to normal modes, with symmetry breaking giving rise to axial and equatorial group local frequencies
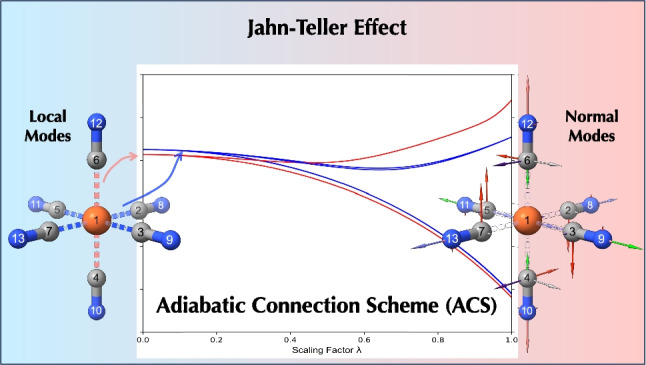

**Supplementary Information:**

The online version contains supplementary material available at 10.1007/s00894-024-05882-8.

## Introduction

The Jahn-Teller (JT) effect leads to a distortion of high-symmetry structures of polyatomic molecules with degenerate electronic states [1–3]. Computational work on the influence of JT and/or PJT effects on the structure and stability of JT systems in both gas phase, solution, and recently also in the solid state [4, 5] include, e.g., monitoring changes in geometry and symmetry [6–8], or changes in molecular properties such as natural bond orbitals (NBO)s and related NBO charges [9]. Also reactivity indices [10] including Fukui functions have been applied to analyze JT distortions [11–15] and dynamic processes such as bond pseudo-rotation triggered by JT distortion have been explored [16–18]. In astrochemistry, investigating structural and electronic changes from associated JT distortion through vibrational spectroscopy of diamondoids has also garnered increasing interest [19–21].

In this work we added another perspective, namely the elucidation of JT distortions based on vibrational spectroscopy. For this purpose, we applied two specific aspects of the local vibrational mode (LVM) theory developed in our group [22, 23], the characterization of normal mode (CNM) procedure [24–27] and an adiabatic connection scheme (ACS) connecting local and normal vibrational modes [22, 23, 28], exploring their potential use for (i) probing JT distortions in transition metal complexes and (ii) subsequently explaining structure and stability of transition metal ion compounds, providing a framework for a new understanding of their structural preferences connected with JT distortions.

**Fig. 1:**
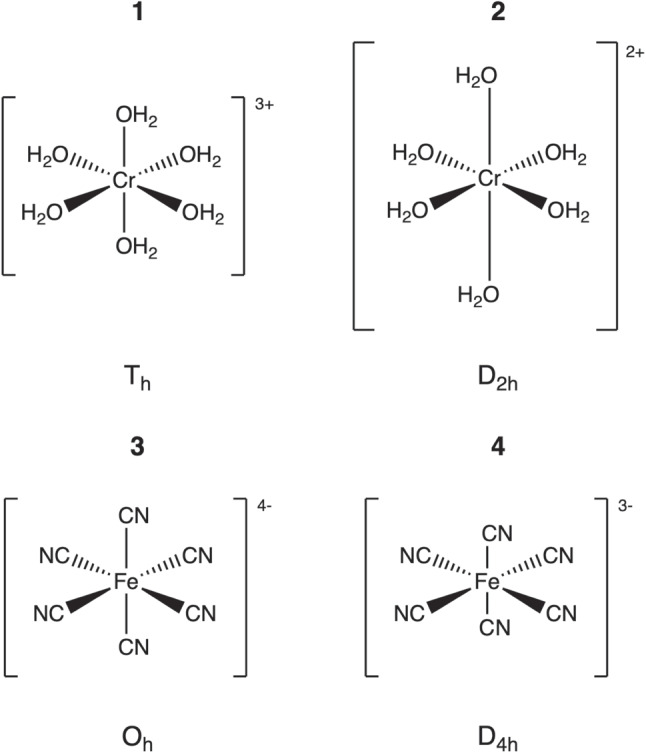
Schematic representation of test examples **1**–**4**, showcasing the point groups associated with the equilibrium geometries obtained at the PBE0/Def2-TZVP level of theory. The difference in axial bond lengths of **2** and **4** has been exaggerated for illustration purposes

We targeted two distinct categories displaying JT distortions, one category with moderate JT distortions accompanied with bond lengthening and one category with slight JT distortions accompanied with bond shortening, often more difficult to capture. As representative for the first category we chose the $\textrm{T}_\textrm{h}$ symmetric hexaaquachromium(III) cation, $[\text {Cr}{(\text {OH}_{2})}_{6}]^{3+}$ (**1** in Fig. 1), and its transition to $[\text {Cr}{(\text {OH}_{2})}_{6}]^{2+}$ with $\textrm{D}_{2\textrm{h}}$ symmetry (**2** in Fig. 1). As representative for the second category we chose ferrous and ferric hexacyanides, $[\text {Fe}{(\text {CN})}_{6}]^{4-}$ with $\textrm{O}_\textrm{h}$ symmetry (**3** in Fig. 1) and its transition to $[\text {Fe}{(\text {CN})}_{6}]^{3-}$ with $\textrm{D}_{4\textrm{h}}$ symmetry (**4** in Fig. 1).

Both hexaaqua complex ions **1** and **2** are high-spin [29], with the JT distortion leading to an elongation of the axial Cr–O bonds [30, 31], see Fig. 1. Conversely, both ferrous and ferric hexacyanides **3** and **4** are low-spin [29]. Neutron diffraction studies have revealed that **3** adopts a regular octahedral configuration $\textrm{O}_\textrm{h}$ [32] whereas for **4**, a $\textrm{d}^{5}$ complex, a slight JT distortion is expected [33, 34]. As confirmed by ab initio calculations, **4** exhibits smaller Fe–ligand $\pi$ back-donation compared to **3** [33]. X-ray diffraction analysis, supported by infrared (IR) spectroscopy and ligand field theoretical calculations, predicts in addition that the iron–ligand bonds in **4** are predominantly of $\sigma$ type, with minimal or no $\pi$ contribution [35]. The **3**/**4** transition stands for an unusual example of JT distortion, and to the best of our knowledge, an explanation of its intricacies is still pending.

Ultimately, this proof-of-concept study was aimed at the investigation of the **1**/**2** case as a commonly known example that exhibits a JT distortion followed by the more difficult **3**/**4** case, comparing common and distinct features. The CNM/ACS protocol for characteristic normal vibrational modes was employed as a novel tool, contributing to a deeper understanding of Jahn-Teller distortions by offering valuable insights into the molecular fragments responsible for symmetry disruption at the molecular level.

## Computational methods

### Geometry optimization and Hessian matrix calculation

All electronic structure calculations were carried out using the Gaussian 16 quantum chemistry program [36] at the density functional theory (DFT) level [37]. An ultra-fine integration grid and a tight convergence criterion were applied for the self-consistent field procedure [38]. The equilibrium geometries of the test examples **1**–**4**, as well as their corresponding Hessian matrices and normal vibrational modes, were obtained using the PBE0 functional [39] in conjunction with the Def2-TZVP basis set [40]. PBE0 has demonstrated favorable outcomes across diverse properties of molecules and materials, encompassing spectroscopic characteristics, making it highly versatile for applications in both quantum chemistry and condensed matter physics [39]. Its performance for transition metal compounds has also been assessed [39]. The hexaaquachromium cations were calculated with unrestricted DFT (UDFT), and ferrous and ferric hexacyanides were calculated with restricted DFT (RDFT) and UDFT, respectively [41].

### Local vibrational mode theory

Normal vibrational modes throughout polyatomic systems are generally delocalized [42, 43], which imposes an important limitation on determining the intrinsic bond strength through the direct use of normal mode frequencies and normal mode force constants. This is precisely where the LVM theory comes into play. LVM was originally introduced by Konkoli and Cremer [44, 45] and further developed by our group. For more details on LVM, including its theoretical foundation and broad application spectrum across chemistry and beyond, the reader is referred to two recent review articles and literature cited therein [22, 23].

Normal vibrational modes $\textbf{d}_{n}$ given in internal coordinates $q_{n}$ (with $n = 1,\cdots ,N_{vib}$ and $N_{vib}$ = (3*N* - 6) for non-linear *N*-atomic complexes and (3*N* - 5) for linear *N*-atomic complexes) and the diagonal normal mode force constant matrix $\textbf{K}$ given in normal coordinates $Q_{n}$ (being available in the output of any modern quantum chemistry software that performs a standard normal mode analysis) are transformed into their local mode counterparts leading to local mode vectors $\textbf{a}_n$ associated with internal coordinates $q_n$ via:

1 $$\begin{aligned} \textbf{a}_n = \frac{\textbf{K}^{-1}\textbf{d}_n^{\dagger }}{\textbf{d}_n\textbf{K}^{-1}\textbf{d}_n^{\dagger }}. \end{aligned}$$

By utilizing the transformation matrix $\textbf{L}$ [42] the local mode vector $\textbf{a}_{n}$ can be easily converted into Cartesian coordinates $\textbf{x}$, resulting in $\textbf{a}_{n}^{x}$ given by the following expression:

2 $$\begin{aligned} \textbf{a}_{n}^{x} = \textbf{L} \textbf{a}_{n}. \end{aligned}$$

The calculation of the corresponding local mode force constant $k_{n}^{a}$ can be performed using the following expression:

3 $$\begin{aligned} k_{n}^{a} = \textbf{a}_{n}^{\dagger } \textbf{K} \textbf{a}_{n}. \end{aligned}$$

This enables the computation of the local mode frequency $\omega ^a_n$:

4 $$\begin{aligned} (\omega ^{a}_{n})^{2} = {(4 \pi ^{2} c^{2})}^{-1} \frac{k_{n}^{a}}{m^{a}_{n}}, \end{aligned}$$

with $m^a_n$ being the local mode mass.

Local mode force constants $k^{a}$ have proven to be a reliable tool to quantify the strength of covalent chemical bonds including metal-ligand bonds and weak chemical interactions such as halogen, chalcogen, pnicogen, tetrel, or hydrogen bonding. For a summary, see Refs. [22, 23] and citations therein. We also derived a new measure for the assessment of metal-ligand bonding, the metal-ligand electronic parameter (MLEP) [46–49], applied, e.g., in recent work for the investigation of iron-ligand bonding in carboxy myoglobins and carboxy neuroglobins [50, 51] and a new assessment of non-covalent $\pi$–interactions in mutated aquomet-myoglobin proteins [52]. Furthermore, our recent incorporation of local mode force constants into lanthanide spectroscopy has provided a novel explanation for the inverse relationship between lanthanide-ligand strength and ligand effective polarizability, offering insights into bonding in lanthanide chemistry [25].

An adiabatic connection scheme (ACS) [28], founded on the Decius compliance matrix [53], denoted as ${\Gamma } = (\textbf{F}^{q})^{-1}$, where $\textbf{F}^{q}$ represents the Hessian matrix in internal coordinates, in conjunction with the Wilson $\textbf{G}$ matrix, establishes a significant one-to-one correspondence between every complete and non-redundant set of local vibrational modes and normal vibrational modes:

5 $$\begin{aligned} ( \textbf{G}_{d} + \lambda \textbf{G}_{od} ) \textbf{R}_{\lambda } = ({\Gamma }_{d} + \lambda {\Gamma }_{od} ) \textbf{R}_{\lambda }{\Lambda }_{\lambda }, \end{aligned}$$

where $\textbf{R} = {\Gamma }^{-1}\textbf{D}$ with $\textbf{D}=\textbf{B}\textbf{L}$ ($\textbf{B}$ is the Wilson B matrix [42]). $\textbf{G}_{d}$ and ${\Gamma }_{d}$, and $\textbf{G}_{od}$ and ${\Gamma }_{od}$ are the diagonal and off-diagonal parts of Decius and Wilson matrices, respectively [42]. The local vibrational modes ($\lambda =0$) are adiabatically transformed into their normal mode counterparts ($\lambda =1$) by a shift parameter $\lambda$ [28].

ACS serves as the fundamental basis for the characterization of normal mode (CNM) procedure [22, 23, 54, 55] which enables the decomposition of each normal vibrational mode $\textbf{l}_{\mu }$ (in Cartesian coordinates) into local vibrational modes contributions. The CNM analysis is conducted for a complete and non-redundant set of $N_{vib}$ local vibrational modes $\textbf{a}_{n}$, offering a powerful tool for quantitatively assessing the composition of normal vibrational modes and facilitating the interpretation of vibrational spectra. The overlapping encoded by $S_{n\mu }$ is [22, 23, 54, 55]

6 $$\begin{aligned} {S}_{n\mu }=\frac{\langle \textbf{a}_{n}^{x} | \textbf{F}^{x} | \textbf{l}_{\mu } \rangle ^2}{\langle \textbf{a}_{n}^{x}| \textbf{F}^{x} |\textbf{a}_{n}^{x}\rangle \langle \textbf{l}_{\mu }| \textbf{F}^{x} |\textbf{l}_{\mu } \rangle }. \end{aligned}$$

Thus, the local mode contribution $C_{n\mu }$ (local mode character) of local vibrational mode $\textbf{a}_{n}^{x}$ to normal vibrational mode $\textbf{l}_{\mu }$ (in Cartesian coordinates) can be calculated as shown below [22, 23, 54, 55]

7 $$\begin{aligned} C_{n\mu } = \frac{S_{n\mu }}{\sum _{m=1}^{N_{vib}}S_{m\mu }}. \end{aligned}$$

Our CNM analysis has facilitated a new way of analyzing and interpreting vibrational spectra stretching from smaller molecular systems to large biomolecules [22, 25–27, 54–58], being described with hybrid quantum chemistry and molecular mechanics (QM/MM) [59–62] thanks to our novel LModeAGen protocol, which automatically determines a complete non-redundant set of local mode parameters [26] and which has been coupled for QM/MM systems with our generalized subsystem vibrational analysis (GSVA), extracting intrinsic fragmental vibrations of any fragment/subsystem from the whole system via the evaluation of the corresponding effective Hessian matrix [63, 64].

**Fig. 2:**
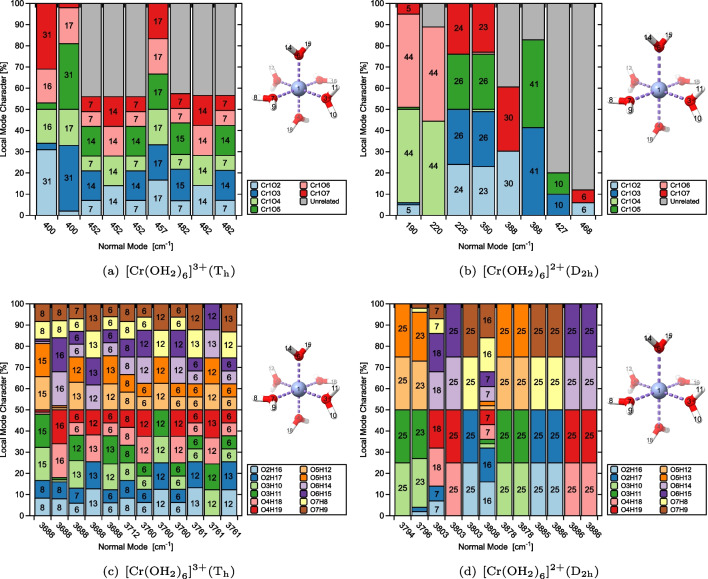
CNM plots capturing metal–ligand (a, b), and O–H (c, d), stretching vibrations for $[\text {Cr}{(\text {OH}_{2})}_{6}]^{3+} (\textrm{T}_{\textrm{h}})$ and $[\text {Cr}{(\text {OH}_{2})}_{6}]^{2+} (\textrm{D}_{2\textrm{h}})$ at the PBE0/Def2-TZVP level of theory

**Fig. 3:**
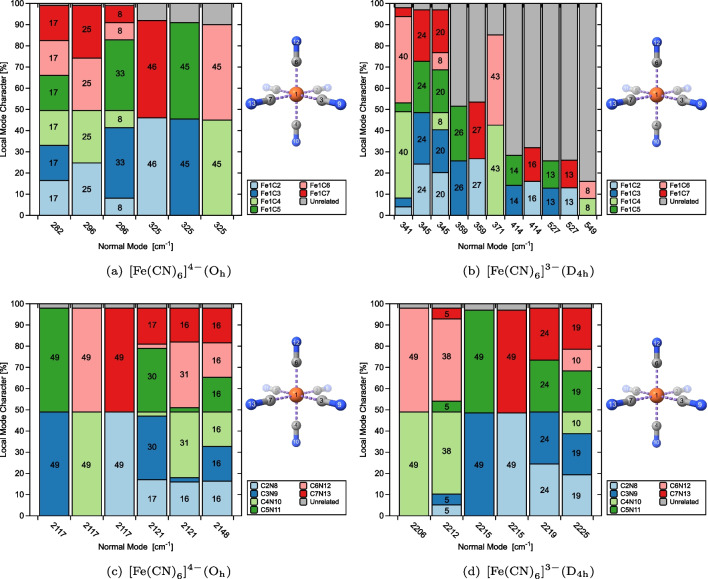
CNM plots capturing metal–ligand (a, b), and C–N (c, d), stretching vibrations for $[\text {Fe}{(\text {CN})}_{6}]^{4-} (\textrm{O}_{\textrm{h}})$ and $[\text {Fe}{(\text {CN})}_{6}]^{3-} (\textrm{D}_{4\textrm{h}})$ at the PBE0/Def2-TZVP level of theory

For the test examples **1**–**4**, the generation of non-redundant and complete sets of local vibrational modes was achieved using the LModeAGen protocol [26]. Subsequently, the local mode analysis was conducted using the standalone LModeA package [65].

### Electron density analysis

The electron density analysis was performed with the AIMAll program [66]. Applying Bader’s quantum theory of atoms in molecules (QTAIM) [67–69], we complemented LVM data with bond properties calculated at the bond critical point $\textbf{r}_{b}$ of the electron density $\rho (\textbf{r})$ between the two atoms forming a chemical bond or weak chemical interaction.

The Cremer-Kraka criterion [70, 71] was applied to assess the covalent character of the chemical bond and/or weak chemical interaction which relies on the local energy density $H(\textbf{r})$ given by the equation:

8 $$\begin{aligned} H(\textbf{r}) = G(\textbf{r}) + V(\textbf{r}) \,, \end{aligned}$$

where $G(\textbf{r})$ is the kinetic energy density (positive, destabilizing), and $V(\textbf{r})$ is the potential energy density (negative, stabilizing). If the value of $H(\textbf{r}_{b})$ is negative the bond/interaction is predominately covalent. Conversely, if it is positive the bond/interaction is primarily electrostatic [70, 71].

## Results and discussion

Figures 2 and 3 provide an overview of the CNM results, displaying the local mode contributions within the characteristic frequency ranges associated with metal–ligand and ligand stretching vibrations for each of the test examples **1**–**4** from Table 1. In the hexaaquachromium complexes, there is an overall increase in the localization of the characteristic normal vibrational modes corresponding to Cr–O and O–H stretching vibrations, as reflected in Fig. 2(a)–(d). In contrast, ferrous and ferric hexacyanides, depicted in Fig. 3(a)–(d), show an opposite trend for Fe–C stretching vibrations. Overall, the normal vibrational modes remain delocalized in both scenarios, as depicted in Figs. 2 and 3. Such delocalization is a characteristic feature observed in systems with significant symmetry, providing valuable insights into the vibrational behavior and the functional group interactions in such compounds. Considering the significance of a reliable bond strength descriptor for understanding coordination sites, the observed delocalization of the characteristic normal vibrational modes analyzed through CNM provides compelling evidence for the utilization of local mode properties to assess the intrinsic strength of metal–ligand bonding in JT systems.

Table 1 presents a comprehensive collection of the properties associated with the local mode parameters of the same metal–ligand and ligand stretching vibrations featured on the CNM plots. The table comprises bond length (*l*), local mode force constant ($k^{a}$), local mode frequency ($\omega ^{a}$), electron density [$\rho (\textbf{r}_{b})$], energy density [$H(\textbf{r}_{b})$], and delocalization index (DI) for the complex ions (test examples **1**–**4**) and the isolated ligands. See Supplementary Information (SI) for Cartesian coordinates of the optimized geometries and the QTAIM atomic charges.

These values were obtained through calculations performed at the PBE0/Def2-TZVP level of theory. On the one hand, the expected equivalence of the properties associated with the local mode parameters of the same metal–ligand and ligand stretching vibrations in both reference complex ions, $[\text {Cr}{(\text {OH}_{2})}_{6}]^{3+} (\textrm{T}_{\textrm{h}})$ and $[\text {Fe}{(\text {CN})}_{6}]^{4-} (\textrm{O}_{\textrm{h}})$, is observed and depicted in Table 1. On the other hand, the breaking of symmetry leads to differentiation between the properties describing the two axial and four equatorial metal–ligand local mode parameters, as observed in the complex ions where JT distortions occur, $[\text {Cr}{(\text {OH}_{2})}_{6}]^{2+} (\textrm{D}_{2\textrm{h}})$ and $[\text {Fe}{(\text {CN})}_{6}]^{3-} (\textrm{D}_{4\textrm{h}})$.

Table 1 shows that the axial local mode parameters, Cr1O4 and Cr1O6, provide insights into the octahedral elongated geometry of $[\text {Cr}{(\text {OH}_{2})}_{6}]^{2+} (\textrm{D}_{2\textrm{h}})$. JT distortion in this compound results in the observed substantial Cr1O4 and Cr1O6 bond length elongation followed by a general reduction in the local force constant, local frequency, electron density, and delocalization index of metal–ligand bond parameters, with the Cr1O4 and Cr1O6 parameters displaying the lowest values. Interestingly, the Cremer-Kraka criterion indicates an increase in axial covalent character and a striking shift to electrostatic character for the equatorial bonds after distortion. On the contrary, due to JT distortion, the local force constant, local frequency, electron density, and delocalization index of water ligands experience a considerable overall increase, with a slightly enhanced covalent character for all the O–H bonds. The results for the isolated form of $\textrm{H}_{2}$O are shown for comparison with the bonded forms, where an increase in bond length and a decrease in the absolute value of all the other computed properties are observed in both cases.

**Table 1:** Selected local mode parameters (param.) followed by the corresponding values of bond length (*l*), local mode force constant ($k^{a}$), local mode frequency ($\omega ^{a}$), electron density [$\rho (\textbf{r}_{b})$], energy density [$H(\textbf{r}_{b})$], and delocalization index (DI), for the complex ions and the isolated ligands, calculated at the PBE0/Def2-TZVP level of theory

	Reference						JT distortion					
	*l*	$k^{a}$	${\omega }^{a}$	$\rho (\textbf{r}_{b})$	$H(\textbf{r}_{b})$	DI	*l*	$k^{a}$	${\omega }^{a}$	$\rho (\textbf{r}_{b})$	$H(\textbf{r}_{b})$	DI
param	[Å]	[mdyn/Å]	[$\text {cm}^{-1}$]	[e/$\text{\AA }^{3}$]	[$\textrm{E}_{\textrm{h}}$/$\text{\AA }^{3}$]		[Å]	[mdyn/Å]	[$\text {cm}^{-1}$]	[e/$\text{\AA }^{3}$]	[$\textrm{E}_{\textrm{h}}$/$\text{\AA }^{3}$]	
	$[\text {Cr}{(\text {OH}_{2})}_{6}]^{3+} (\textrm{T}_{\textrm{h}})$						$[\text {Cr}{(\text {OH}_{2})}_{6}]^{2+} (\textrm{D}_{2\textrm{h}})$					
Cr1O2	1.997	1.611	473	0.526	$-$0.011	0.42	2.091	0.822	338	0.388	0.018	0.32
Cr1O3	1.997	1.611	473	0.526	$-$0.011	0.42	2.087	0.868	347	0.393	0.017	0.32
Cr1O4	1.997	1.611	473	0.526	$-$0.011	0.42	2.374	0.335	216	0.236	$-$0.019	0.20
Cr1O5	1.997	1.611	473	0.526	$-$0.011	0.42	2.087	0.868	347	0.393	0.017	0.32
Cr1O6	1.997	1.611	473	0.526	$-$0.011	0.42	2.374	0.335	216	0.236	$-$0.019	0.20
Cr1O7	1.997	1.611	473	0.526	$-$0.011	0.42	2.091	0.822	338	0.388	0.018	0.32
O2H16	0.973	7.720	3717	2.283	$-$4.459	0.51	0.964	8.219	3836	2.374	$-$4.612	0.57
O2H17	0.973	7.720	3717	2.283	$-$4.459	0.51	0.964	8.219	3836	2.374	$-$4.612	0.57
O3H10	0.973	7.720	3717	2.283	$-$4.459	0.51	0.965	8.182	3827	2.367	$-$4.601	0.56
O3H11	0.973	7.720	3717	2.283	$-$4.459	0.51	0.965	8.182	3827	2.367	$-$4.601	0.56
O4H18	0.973	7.720	3717	2.283	$-$4.459	0.51	0.964	8.196	3830	2.390	$-$4.606	0.60
O4H19	0.973	7.720	3717	2.283	$-$4.459	0.51	0.964	8.196	3830	2.390	$-$4.606	0.60
O5H12	0.973	7.720	3717	2.283	$-$4.459	0.51	0.965	8.182	3827	2.367	$-$4.601	0.56
O5H13	0.973	7.720	3717	2.283	$-$4.459	0.51	0.965	8.182	3827	2.367	$-$4.601	0.56
O6H14	0.973	7.720	3717	2.283	$-$4.459	0.51	0.964	8.196	3830	2.390	$-$4.606	0.60
O6H15	0.973	7.720	3717	2.283	$-$4.459	0.51	0.964	8.196	3830	2.390	$-$4.606	0.60
O7H8	0.973	7.720	3717	2.283	$-$4.459	0.51	0.964	8.219	3836	2.374	$-$4.612	0.57
O7H9	0.973	7.720	3717	2.283	$-$4.459	0.51	0.964	8.219	3836	2.374	$-$4.612	0.57
	$$\textrm{H}_{2}\textrm{O} (\textrm{C}_{2 \textrm{v}})$$											
O1H2	0.960	8.422	3883	2.456	$-$4.652	0.68						
	$[\text {Fe}{(\text {CN})}_{6}]^{4-} (\textrm{O}_{\textrm{h}})$						$[\text {Fe}{(\text {CN})}_{6}]^{3-} (\textrm{D}_{4\textrm{h}})$					
Fe1C2	1.992	1.062	427	0.625	$-$0.168	0.70	1.976	1.529	513	0.710	$-$0.252	0.66
Fe1C3	1.992	1.062	427	0.625	$-$0.168	0.70	1.976	1.529	513	0.710	$-$0.252	0.66
Fe1C4	1.992	1.062	427	0.625	$-$0.168	0.70	1.967	1.495	507	0.704	$-$0.243	0.70
Fe1C5	1.992	1.062	427	0.625	$-$0.168	0.70	1.976	1.529	513	0.710	$-$0.252	0.66
Fe1C6	1.992	1.062	427	0.625	$-$0.168	0.70	1.967	1.495	507	0.704	$-$0.243	0.70
Fe1C7	1.992	1.062	427	0.625	$-$0.168	0.70	1.976	1.529	513	0.710	$-$0.252	0.66
C2N8	1.174	17.030	2115	3.236	$-$6.351	2.11	1.163	18.342	2195	3.295	$-$6.498	2.20
C3N9	1.174	17.030	2115	3.236	$-$6.351	2.11	1.163	18.342	2195	3.295	$-$6.498	2.20
C4N10	1.174	17.030	2115	3.236	$-$6.351	2.11	1.164	18.240	2189	3.289	$-$6.483	2.18
C5N11	1.174	17.030	2115	3.236	$-$6.351	2.11	1.163	18.342	2195	3.295	$-$6.498	2.20
C6N12	1.174	17.030	2115	3.236	$-$6.351	2.11	1.164	18.240	2189	3.289	$-$6.483	2.18
C7N13	1.174	17.030	2115	3.236	$-$6.351	2.11	1.163	18.342	2195	3.295	$-$6.498	2.20
	$$\text {CN}^{-} (\textrm{C}_{\infty \textrm{v}})$$											
N1C2	1.170	17.770	2160	3.291	$-$6.487	2.35						

**Fig. 4:**
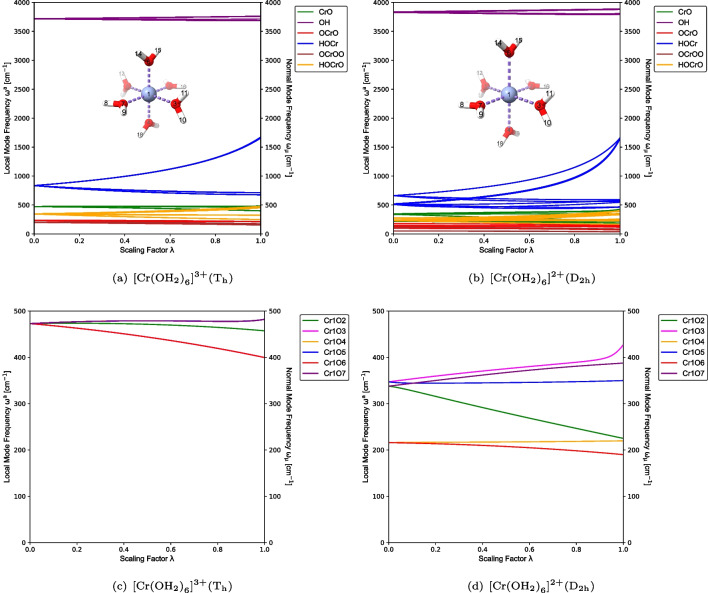
ACS plots relating the frequencies of a complete and non-redundant set of local modes (left) with those of normal modes (right) for $[\text {Cr}{(\text {OH}_{2})}_{6}]^{3+} (\textrm{T}_{\textrm{h}})$ (a), and $[\text {Cr}{(\text {OH}_{2})}_{6}]^{2+} (\textrm{D}_{2\textrm{h}})$ (b). The pairwise breakdown of the corresponding separate metal–ligand frequency range is provided on the bottom (c, d). Results obtained at the PBE0/Def2-TZVP level of theory

In the case of $[\text {Fe}{(\text {CN})}_{6}]^{3-} (\textrm{D}_{4\textrm{h}})$, Table 1 also shows that Fe1C4 and Fe1C6 local stretching modes serve as indicators of the octahedral compressed geometry. Despite the subtle compression, there is a significant overall increase in the local force constant, local frequency, and electron density for both metal–ligand and ligand $\text {CN}^{-}$ bond parameters, with Fe1C4 and Fe1C6 parameters showing slightly smaller values compared to the equatorial local mode parameters. Additionally, the energy density values demonstrate that both metal–ligand and ligand bonds become notably more covalent upon distortion, with no shift in bond nature. It has to be noted that the bond length is not always a qualified bond strength descriptor. Numerous cases have been reported in the literature illustrating that a shorter bond is not always a stronger bond [72–78]. This also applies to the metal–ligand bonds in $[\text {Fe}{(\text {CN})}_{6}]^{3-} (\textrm{D}_{4\textrm{h}})$, despite the delocalization index indicating otherwise.

Comparing the isolated and bonded forms of the $\text {CN}^{-}$ ligand reveals that in $[\text {Fe}{(\text {CN})}_{6}]^{4-} (\textrm{O}_{\textrm{h}})$ a slight increase in bond length and a decrease in the local force constant, local frequency, electron density, the absolute value of energy density, and delocalization index occur as expected due to the $\pi$ back-donation phenomenon discussed in the literature [33] (see Table 1 for details). When compared to $[\text {Fe}{(\text {CN})}_{6}]^{3-} (\textrm{D}_{4\textrm{h}})$, both the electron and energy density change slightly, especially for the axial ligands, with a slight decrease in bond length. Still, there is an increase in both the local force constant and local frequency upon coordination with Fe(III). The weaker back-donation effect found in $[\text {Fe}{(\text {CN})}_{6}]^{3-}$ can account for this behavior, as it implies a reduced transfer of electron density from the metal center to the ligands, affecting the CN bond characteristics. The difference in vibrational properties between $[\text {Fe}{(\text {CN})}_{6}]^{4-} (\textrm{O}_{\textrm{h}})$ and $[\text {Fe}{(\text {CN})}_{6}]^{3-} (\textrm{D}_{4\textrm{h}})$ is in accordance with the literature [33, 79].

**Fig. 5:**
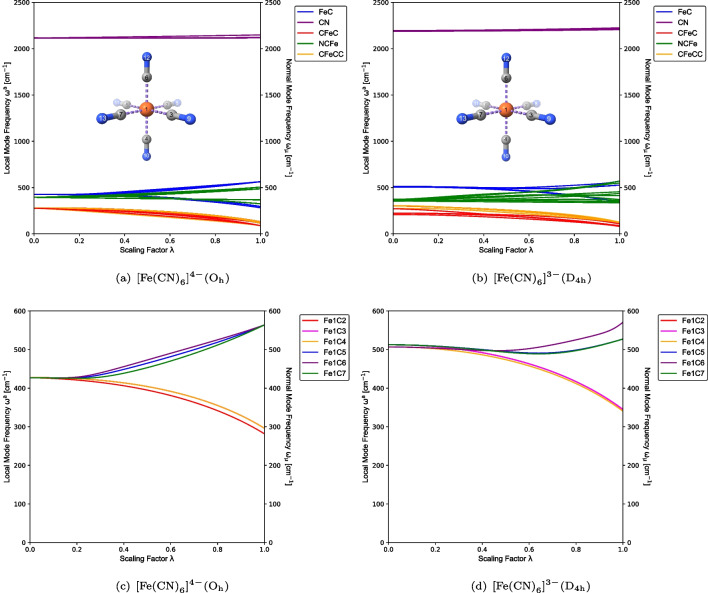
ACS plots relating the frequencies of a complete and non-redundant set of local modes (left) with those of normal modes (right) for $[\text {Fe}{(\text {CN})}_{6}]^{4-} (\textrm{O}_{\textrm{h}})$ (a), and $[\text {Fe}{(\text {CN})}_{6}]^{3-} (\textrm{D}_{4\textrm{h}})$ (b). The pairwise breakdown of the corresponding separate metal–ligand frequency range is provided on the bottom (c, d). Results obtained at the PBE0/Def2-TZVP level of theory

Furthermore, Figs. 4 and 5 present the ACS plots for test examples **1**–**4**. In these plots, the parameter $\lambda$ distinguishes between local modes ($\lambda =0$) and normal modes ($\lambda =1$). Both $[\text {Cr}{(\text {OH}_{2})}_{6}]^{3+}$ and $[\text {Cr}{(\text {OH}_{2})}_{6}]^{2+}$ exhibit 51 normal vibrational modes, which are adiabatically connected to 6 groups of local vibrational modes, as shown in Fig. 4. Similarly, $[\text {Fe}{(\text {CN})}_{6}]^{4-}$ and $[\text {Fe}{(\text {CN})}_{6}]^{3-}$ possess 33 normal vibrational modes, which are adiabatically connected to 5 groups of local vibrational modes, as depicted in Fig. 5.

From Fig. 4(a) and (c), it is evident that the $[\text {Cr}{(\text {OH}_{2})}_{6}]^{3+}$ complex ion with the $\textrm{T}_{\textrm{h}}$ point group symmetry exhibits equivalence among the $\text {OH}_{2}$ ligands, leading to the expected result. Consequently, several degenerate local vibrational modes can be observed, which can be categorized as follows: six degenerate Cr–O bond stretching modes and twelve degenerate O–H bond stretching modes, as highlighted in Table 1, in addition to six degenerate local O–Cr–O modes, twelve degenerate local H–O–Cr modes, three degenerate local O–Cr–O–O modes, and twelve degenerate local H–O–Cr–O modes. It can be observed from Table 1 and Fig. 4(c) and (d) that the expected sixfold degeneracy of the Cr–O bond local vibrational modes in $[\text {Cr}{(\text {OH}_{2})}_{6}]^{3+}$ is broken, forming three groups of twofold degeneracy in $[\text {Cr}{(\text {OH}_{2})}_{6}]^{2+}$. Figure 4(b) and (d) clearly illustrate the symmetry breaking due to the JT elongation captured by ACS. The axial local vibrational modes, denoted by Cr1O6 and Cr1O4, occur as the lower-frequency twofold degenerate group, whereas the four equatorial local vibrational modes split into two very closely separated groups due to the equilibrium geometry being a $\textrm{D}_{2\textrm{h}}$ minimum. Of particular importance is the local vibrational mode evidence of the strength of the JT distortion, seen by the pronounced frequency separation between the axial and equatorial groups of local vibrational modes in Fig. 4(d). This finding agrees with the marked difference in bond length tabulated in Table 1.

It is worth mentioning that crystallographic data and EPR studies have indicated that $[\text {Cr}{(\text {OH}_{2})}_{6}]^{2+}$ preferably exhibits an octahedral elongated structure [30, 31]. Furthermore, it is well-established [6, 29, 31] that octahedral JT elongated structures can be understood in terms of the d-electron stabilization energy, where a greater electrostatic repulsion is observed between the metal ion and the two ligands along the z-axis, leading to their stabilizing elongation. It is also remarkable that the local mode force constants ($k^a$) are smaller for Cr1O4 and Cr1O6 bonds, indicating that they are weaker than the Cr–O equatorial bonds. This trend in $k^a$ can be attributed to the elongation of the axial Cr–O bonds, which serves to decrease the repulsion between the d-orbitals along the z-axis and the ligands. By elongating the axial bonds, the d-electron distribution around the chromium ion is modified, leading to reduced energy levels and increased stability. This effect is in line with the concept of JT elongation, where the distortion of the octahedral geometry results in lower energy states for the system. Moreover, the observed decrease in $\rho (\textbf{r}_b)$ upon JT distortion further supports the weakening of the axial bonds. Additionally, the negative value of $H(\textbf{r}_b)$ for Cr–O axial bonds reveals that the electron density at the bond critical point is still significant, indicating a considerable bonding interaction despite the weakening. Overall, the findings reported in this study align with the known principles of JT elongation in octahedral complexes and provide valuable insights into the structural and electronic properties of $[\text {Cr}{(\text {OH}_{2})}_{6}]^{2+}$.

Figure 5(a) and (c) provide clear evidence that the $[\text {Fe}{(\text {CN})}_{6}]^{4-}$ complex ion, exhibiting $\textrm{O}_{\textrm{h}}$ point group symmetry, demonstrates equivalence among the $\text {CN}$ ligands, as expected. Consequently, several degenerate local vibrational modes can be observed, classified as follows: six degenerate Fe–C bond stretching modes and six degenerate C–N bond stretching modes, as shown in Table 1, in addition to six degenerate local C–Fe–C modes, twelve degenerate local N–C–Fe modes, and three degenerate local C–Fe–C–C modes. From Table 1 and Fig. 5(c) and (d), it can be observed that the sixfold degeneracy of the Fe–C bond local vibrational modes in $[\text {Fe}{(\text {CN})}_{6}]^{4-}$ is broken in $[\text {Fe}{(\text {CN})}_{6}]^{3-}$, resulting in two groups of twofold and fourfold degeneracy. The symmetry breaking due to the JT compression captured by ACS is clearly illustrated in Fig. 5(b) and (d).

The axial local vibrational modes, denoted by Fe1C6 and Fe1C4, occur as the lower-frequency twofold degenerate group, while the four equatorial local vibrational modes occur as the fourfold degenerate group, in line with the equilibrium geometry of $\textrm{D}_{4\textrm{h}}$ symmetry. The local vibrational mode evidence of the weakness of the JT distortion in $[\text {Fe}{(\text {CN})}_{6}]^{3-}$, as mentioned in the literature [33], can be observed by the small frequency separation between the axial and equatorial groups of local vibrational modes in Fig. 5(d). This finding is in agreement with the minor difference in bond length tabulated in Table 1.

As an important development, the innovative method of probing JT distortions through ACS and CNM, as demonstrated in this study, is poised to undergo further expansion to encompass the vibrational analysis of coordination compounds with diverse sizes and complexities. This advancement opens up a wide range of potential applications that can be tailored to investigate different metal ions, ligands, chemical environments, and coordination spheres, thereby enriching our understanding of coordination chemistry. Work is in progress to extend the protocol described herein to post-Hartree-Fock methods.

## Conclusions

We developed in this work a new way for assessing JT distortions via a combination of two important features of LVM, namely the characterization of normal mode (CNM) procedure and an adiabatic connection scheme (ACS) connecting local and normal vibrational modes. The viability of this new CNM/ACS protocol was successfully demonstrated through the examination of classical coordination compounds at the PBE0/Def2-TZVP level of theory. Our CNM/ACS results provide new insights into the changes in symmetry resulting from JT distortions, which sets the stage for applying this approach to a diverse array of systems with varying size and complexity. More specifically, the following results emerged from our study, unravelling (i) distinct pairwise patterns of ACS and CNM variations across different oxidation states, and (ii) subtle differences between systems **1**/**2**, representing the category with moderate JT distortions accompanied with bond lengthening, and **3**/**4**, representing the category with slight JT distortions accompanied with bond shortening:

- CNM results illustrated that the characteristic normal vibrational modes exhibit overall delocalization for both scenarios **1**/**2** and **3**/**4**, confirming what we generally observe for systems with high symmetry. There is a slight increase in localization in the transition from **1** to **2** for both Cr–O and O–H stretching vibrations. In contrast, there is a reverse trend for Fe–C stretching vibrations in the transition from **3** to **4**.
- The ACS analysis clearly reflected the symmetry breaking via the differentiation between axial and equatorial groups following redox changes for the pairs **1**/**2** and **3**/**4** as a consequence of the JT distortions. The ACS profile of **4** exhibits a slight frequency separation between the axial and equatorial groups, whereas in the case of **2**, a more pronounced distinction is observed. This is in accordance with the fact that **2** undergoes a moderate JT distortion compared to **4**, which experiences a minor JT distortion. These findings suggest the use of ACS patterns as sensitive indicators of the extent of JT distortions, based on the frequency separation between axial and equatorial groups displayed in the ACS profile.
- The Cremer-Kraka criterion revealed an enhancement in axial covalent nature and a noticeable shift toward electrostatic character for the equatorial bonds following distortion from **1** to **2**. Moving from **3** to **4**, the energy density data indicate a significant increase in covalence for both metal–ligand and ligand bonds upon distortion, while their bond nature remains consistent. Comparison with the isolated $\text {CN}^{-}$ molecule confirms the distinct $\pi$ back-donation disparity between **3** and **4**, as documented in the literature [32–35].

In summary, the ACS profile emerges as a powerful tool to investigate and confirm the subtle and less-explored JT distortion in **4** and similar systems, often more difficult to capture, and in this way providing an answer to the current debate on the existence of a JT distortion in this system. As demonstrated in this proof-of-concept study our new CNM/ACS protocol opens up new possibilities for future JT studies in coordination chemistry. Its adaptability to different metal ions, ligands, chemical environments, and coordination spheres can be utilized to gain a better understanding of coordination compounds. The versatility of this innovative approach allows for a broad range of applications, opening the avenue for further CNM/ACS JT explorations stretching into organic chemistry [80], biochemistry [81] and beyond [82, 83]. Work is in progress to extend the protocol described herein to post-Hartree-Fock methods.

### Supplementary Information

Below is the link to the electronic supplementary material.

Supplementary file 1 (pdf 129 KB)

## Data Availability

No datasets were generated or analyzed during the current study.

## References

[1] Jahn HA, Teller E (1937) Stability of polyatomic molecules in degenerate electronic states. I. Orbital degeneracy. Proc R Soc Lond A 161:220–235 10.1098/rspa.1937.0142

[2] Liehr AD (1963) Topological aspects of the conformational stability problem. Part I Non-degenerate electronic states. J Chem Phys 67:389–471 10.1021/j100796a043

[3] Liehr AD (1963) Topological aspects of the conformational stability problem. Part II Non-degenerate electronic states. J Chem Phys 67:471–494 10.1021/j100796a044

[4] Pascale F, D’Arco P, Lebègue S, Dovesi R (2024) Jahn-Teller distortion, octahedra rotations and orbital ordering in perovskites: KScF as a model system. J Comput Chem. 10.1002/jcc.27306 38217380 10.1002/jcc.27306

[5] Kim WJ, Smeaton MA, Jia C, Goodge BH, Byeong-Gwan Lee K, Osada M, Jost D, Levlev AV, Moritz B, Kourkoutis LF, Devereaux TP, Hwang HY (2023) Geometric frustration of Jahn-Teller order in the infinite-layer lattice. Nature 615:237 36813969 10.1038/s41586-022-05681-2

[6] Conradie J (2019) Jahn-Teller effect in high spin d4 and d9 octahedral metal-complexes. Inorg Chim Acta 486:193–199 10.1016/j.ica.2018.10.040

[7] Da-yang TE, Fifen JJ, Malloum A, Lahmar S, Nsangou M, Conradie J (2020) Structures of the solvated copper(II) ion in ammonia at various temperatures. New J Chem 44:3637–3653 10.1039/C9NJ05169D

[8] Conradie J (2024) Effect of density functional approximations on the calculated Jahn-Teller distortion in bis(terpyridine)manganese(III) and related compounds. J Mol Model 30:20 38165497 10.1007/s00894-023-05812-0 PMC10761540

[9] Kouchakzadeh G, Mahmoudzadeh G (2023) The Pseudo Jahn-Teller effect and NBO analysis for untangling the symmetry breaking in the planar configurations of MX (M=Si, Ge and X=Cl, Br, I): effect on electronic structure and chemical properties. J Mol Model 30:1 38052766 10.1007/s00894-023-05792-1

[10] Ayers PW (2001) Strategies for computing chemical reactivity indices. Theor Chem Acc 106:271–279 10.1007/PL00012385

[11] Oller J, Jaque P (2023) Connection between nuclear and electronic Fukui functions beyond frontier molecular orbitals. J Chem Phys 159:124112 38127388 10.1063/5.0169403

[12] Gómez S, Rojas-Valencia N, Toro-Labbé A, Restrepo A (2023) The transition state region in nonsynchronous concerted reactions. J Chem Phys 158:084109 36859077 10.1063/5.0133487

[13] Toro-Labbé A, Gutiérrez-Oliva S, Murray JS, Politzer P (2007) A new perspective on chemical and physical processes: the reaction force. Mol Phys 105:2619–2625 10.1080/00268970701604663

[14] Morel C, Grand A, Toro-Labbé A (2005) New dual descriptor for chemical reactivity. J Phys Chem A 109:205–212 16839107 10.1021/jp046577a

[15] Balawender R, De Proft F, Geerlings P (2001) Nuclear Fukui function and Berlin’s binding function: prediction of the Jahn-Teller distortion. J Chem Phys 114:4441–4449 10.1063/1.1346579

[16] Zou W, Cremer D (2014) Description of bond pseudorotation, bond pseudolibration, and ring pseudoinversion processes caused by the pseudo-Jahn-Teller effect: fluoro derivatives of the cyclopropane radical cation. Aust J Chem 67:435 10.1071/CH13480

[17] Zou W, Filatov M, Cremer D (2012) Bond pseudorotation, Jahn-Teller, and pseudo-Jahn-Teller effects in the cyclopentadienyl cation and its pentahalogeno derivatives. Int J Quantum Chem 112:3277–3288 10.1002/qua.24116

[18] Zou W, Izotov D, Cremer D (2011) New way of describing static and dynamic deformations of the Jahn-Teller type in ring molecules. J Phys Chem A 115:8731–8742 21736381 10.1021/jp2041907

[19] Patzer A, Schütz M, Möller T, Dopfer O (2012) Infrared spectrum and structure of the adamantane cation: direct evidence for Jahn-Teller distortion. Angew Chem Int Ed 51:4925–4929 10.1002/anie.201108937 22287542

[20] George MAR, Förstel M, Dopfer O (2020) Infrared spectrum of the adamantane–water cation: hydration-induced C-H bond activation and free internal water rotation. Angew Chem Int Ed 59:12098–12104 10.1002/anie.202003637 PMC7383494 32392402

[21] George MAR, Dopfer O (2022) Infrared spectrum of the amantadine cation: opening of the diamondoid cage upon ionization. J Phys Chem Lett 13:449–454 34990124 10.1021/acs.jpclett.1c03948

[22] Kraka E, Quintano M, Force HWL, Antonio JJ, Freindorf M (2022) The local vibrational mode theory and its place in the vibrational spectroscopy arena. J Phys Chem A 126:8781–8900 36346943 10.1021/acs.jpca.2c05962

[23] Kraka E, Zou W, Tao Y (2020) Decoding chemical information from vibrational spectroscopy data: local vibrational mode theory. WIREs: Comput Mol Sci 10:1480

[24] Verma N, Tao Y, Zou W, Chen X, Chen X, Freindorf M, Kraka E (2020) A critical evaluation of Vibrational Stark Effect (VSE) probes with the local vibrational mode theory. Sensors 20:2358 32326248 10.3390/s20082358 PMC7219233

[25] Moura Jr RT, Quintano M, Santos-Jr CV, Albuquerque VACA, Aguiar EC, Kraka E, Carneiro Neto AN (2022) Featuring a new computational protocol for the estimation of intensity and overall quantum yield in lanthanide chelates with applications to Eu(III) mercapto-triazole Schiff base ligands. Optical Materials: X 16:100216–110021615

[26] Moura Jr RT, Quintano M, Antonio JJ, Freindorf M, Kraka E (2022) Automatic generation of local vibrational mode parameters: from small to large molecules and QM/MM systems. J Phys Chem A 126:9313–9331 10.1021/acs.jpca.2c07871 36472412

[27] Quintano M, Delgado AAA, Moura Jr RT, Freindorf M, Kraka E (2022) Local mode analysis of characteristic vibrational coupling in nucleobases and Watson–Crick base pairs of DNA. Electron Struct 4(12):044005–104400517

[28] Zou W, Kalescky R, Kraka E, Cremer D (2012) Relating normal vibrational modes to local vibrational modes with the help of an adiabatic connection scheme. J Chem Phys 137:084114 22938225 10.1063/1.4747339

[29] Housecroft CE, Sharpe AG (2012) Inorganic chemistry, 4th edn. Pearson, Essex

[30] Telser J, Pardi LA, Krzystek J, Brunel L-C (1998) EPR spectra from “EPR-Silent’’ species: high-field EPR spectroscopy of aqueous Chromium(II). Inorg Chem 37:5769–5775 10.1021/ic9806683 12184765

[31] Conradie J (2018) Structural and electronic data of three first-row transition octahedral hexaaquametal(II) ions, metal=Cr, Ni or Cu. Data in Brief 21:2051–2058 30533451 10.1016/j.dib.2018.11.055 PMC6262161

[32] Taylor JC, Mueller MH, Hitterman RL (1970) A neutron diffraction study of ferroelectric KFCT, KFe(CN).3DO, above the Curie temperature. Acta Crystallogr A 26:559–567 10.1107/S0567739470001407

[33] Kunnus K, Zhang W, Delcey MG, Pinjari RV, Miedema PS, Schreck S, Quevedo W, Schröder H, Föhlisch A, Gaffney KJ, Lundberg M, Odelius M, Wernet P (2016) Viewing the valence electronic structure of ferric and ferrous hexacyanide in solution from the Fe and cyanide perspectives. J Phys Chem B 120:7182–7194 27380541 10.1021/acs.jpcb.6b04751

[34] Atanasov M, Comba P, Daul CA, Hauser A (2007) DFT-based studies on the Jahn-Teller effect in 3D hexacyanometalates with orbitally degenerate ground states. J Phys Chem A 111:9145–9163 17718456 10.1021/jp0731912

[35] Vannerberg N-G, Pedersen E, Romanowska E, Rudén U, Pilotti Å (1972) The OD structures of KFe(CN) and KCo(CN). Acta Chem Scand 26:2863–2876 10.3891/acta.chem.scand.26-2863

[36] Frisch MJ, Trucks GW, Schlegel HB, Scuseria GE, Robb MA, Cheeseman JR, Scalmani G, Barone V, Petersson GA, Nakatsuji H, Li X, Caricato M, Marenich AV, Bloino J, Janesko BG, Gomperts R, Mennucci B, Hratchian HP, Ortiz JV, Izmaylov AF, Sonnenberg JL, Williams-Young D, Ding F, Lipparini F, Egidi F, Goings J, Peng B, Petrone A, Henderson T, Ranasinghe D, Zakrzewski VG, Gao J, Rega N, Zheng G, Liang W, Hada M, Ehara M, Toyota K, Fukuda R, Hasegawa J, Ishida M, Nakajima T, Honda Y, Kitao O, Nakai H, Vreven T, Throssell K, Montgomery JA Jr, Peralta JE, Ogliaro F, Bearpark MJ, Heyd JJ, Brothers EN, Kudin KN, Staroverov VN, Keith TA, Kobayashi R, Normand J, Raghavachari K, Rendell AP, Burant JC, Iyengar SS, Tomasi J, Cossi M, Millam JM, Klene M, Adamo C, Cammi R, Ochterski JW, Martin RL, Morokuma K, Farkas O, Foresman JB, Fox DJ (2016) Gaussian 16 Revision C.01. Gaussian Inc. Wallingford CT

[37] Parr RG, Yang W (1989) Density-functional theory of atoms and molecules. Oxford University Press, New York

[38] Gräfenstein J, Cremer D (2007) Efficient density-functional theory integrations by locally augmented radial grids. J Chem Phys 127(16):164113 17979325 10.1063/1.2794038

[39] Adamo C, Barone V (1999) Toward reliable density functional methods without adjustable parameters: The PBE0 model. J Chem Phys 110:6158–6170 10.1063/1.478522

[40] Weigend F, Ahlrichs R (2005) Balanced basis sets of split valence, triple zeta valence and quadruple zeta valence quality for H to Rn: design and assessment of accuracy. Phys Chem Chem Phys 7:3297–3305 16240044 10.1039/b508541a

[41] Wang J, Becke AD, Vedene H, Smith J (1995) Evaluation of in restricted, unrestricted Hartree–Fock, and density functional based theories. J Chem Phys 102:3477–3480 10.1063/1.468585

[42] Wilson EB, Decius JC, Cross PC (1955) Molecular vibrations: the theory of infrared and Raman vibrational spectra. McGraw-Hill, New York

[43] Wilson EB (1941) Some mathematical methods for the study of molecular vibrations. J Chem Phys 9:76–84 10.1063/1.1750829

[44] Konkoli Z, Cremer D (1998) A new way of analyzing vibrational spectra. I. Derivation of adiabatic internal modes. Int J Quantum Chem 67:1–9 10.1002/(SICI)1097-461X(1998)67:1<1::AID-QUA1>3.0.CO;2-Z

[45] Konkoli Z, Larsson JA, Cremer D (1998) A new way of analyzing vibrational spectra. II. Comparison of internal mode frequencies. Int J Quantum Chem 67:11–27 10.1002/(SICI)1097-461X(1998)67:1<11::AID-QUA2>3.0.CO;2-1

[46] Kraka E, Freindorf M (2020) Characterizing the metal ligand bond strength via vibrational spectroscopy: the Metal Ligand Electronic Parameter (MLEP). In: Lledós A, Ujaque G (eds) Topics in organometallic chemistry - new directions in the modeling of organometallic reactions, vol 67. Springer, eBook, pp 1–43

[47] Cremer D, Kraka E (2017) Generalization of the Tolman electronic parameter: the metal-ligand electronic parameter and the intrinsic strength of the metal-ligand bond. Dalton Trans 46:8323–8338 28350024 10.1039/C7DT00178A

[48] Setiawan D, Kalescky R, Kraka E, Cremer D (2016) Direct measure of metal-ligand bonding replacing the Tolman electronic parameter. Inorg Chem 55:2332–2344 26900632 10.1021/acs.inorgchem.5b02711

[49] Kalescky R, Kraka E, Cremer D (2013) New approach to Tolman’s electronic parameter based on local vibrational modes. Inorg Chem 53:478–495 24320732 10.1021/ic4024663

[50] Freindorf M, Kraka E (2020) Critical assessment of the FeC and CO bond strength in carboxymyoglobin - A QM/MM local vibrational mode study. J Mol Model 26:281–128115 32970192 10.1007/s00894-020-04519-w

[51] Freindorf M, Delgado AAA, Kraka E (2022) CO bonding in hexa– and pentacoordinate carboxy–neuroglobin – A QM/MM and local vibrational mode study. J Comp Chem 43:1725–1746 10.1002/jcc.26973

[52] Antonio JJ, Kraka E (2023) Non-covalent –interactions in mutated aquomet-Myoglobin proteins: a QM/MM and local vibrational mode study. Biochemistry 62:2325–2337 37458402 10.1021/acs.biochem.3c00192

[53] Decius JC (1963) Compliance matrix and molecular vibrations. J Chem Phys 38:241–248 10.1063/1.1733469

[54] Konkoli Z, Cremer D (1998) A new way of analyzing vibrational spectra. III. Characterization of normal vibrational modes in terms of internal vibrational modes. Int J Quantum Chem 67:29–40 10.1002/(SICI)1097-461X(1998)67:1<29::AID-QUA3>3.0.CO;2-0

[55] Konkoli Z, Larsson JA, Cremer D (1998) A new way of analyzing vibrational spectra. IV. Application and testing of adiabatic modes within the concept of the characterization of normal modes. Int J Quantum Chem 67:41–55 10.1002/(SICI)1097-461X(1998)67:1<41::AID-QUA4>3.0.CO;2-Z

[56] Cremer D, Larsson JA, Kraka E (1998) New developments in the analysis of vibrational spectra on the use of adiabatic internal vibrational modes. In: Parkanyi C (ed) Theoretical and computational chemistry. Elsevier, Amsterdam, pp 259–327

[57] Quintano M, Kraka E (2022) Theoretical insights into the linear relationship between pK values and vibrational frequencies. Chem Phys Lett 803:139746–11397467 10.1016/j.cplett.2022.139746

[58] Quintano M, Moura Jr RT, Kraka E (2023) The pK rule in light of local mode force constants. Chem Phys Lett 826:140654–11406547

[59] Kamp MW, Mulholland AJ (2013) Combined quantum mechanics/molecular mechanics (QM/MM) methods in computational enzymology. Biochemistry 52(16):2708–2728 23557014 10.1021/bi400215w

[60] Tzeliou CE, Mermigki MA, Tzeli D (2022) Review on the QM/MM methodologies and their application to metalloproteins. Molecules 27(9):2660 35566011 10.3390/molecules27092660 PMC9105939

[61] Chung LW, Sameera WMC, Ramozzi R, Page AJ, Hatanaka M, Petrova GP, Harris TV, Li X, Ke Z, Liu F, Li H-B, Ding L, Morokuma K (2015) The ONIOM method and its applications. Chem Rev 115:5678–5796 25853797 10.1021/cr5004419

[62] Warshel A, Levitt M (1976) Theoretical studies of enzymic reactions: dielectric, electrostatic and steric stabilization of the carbonium ion in the reaction of lysozyme. J Mol Biol 103(2):227–249 985660 10.1016/0022-2836(76)90311-9

[63] Tao Y, Zou W, Nanayakkara S, Freindorf M, Kraka E (2021) A revised formulation of the generalized subsystem vibrational analysis (GSVA). Theor Chem Acc 140:31–1315 33716564 10.1007/s00214-021-02727-y PMC7942689

[64] Tao Y, Tian C, Verma N, Zou W, Wang C, Cremer D, Kraka E (2018) Recovering intrinsic fragmental vibrations using the generalized subsystem vibrational analysis. J Chem Theory Comput 14:2558–2569 29634270 10.1021/acs.jctc.7b01171

[65] Zou W, Moura Jr RT, Quintano M, Bodo F, Tao Y, Freindorf M, Makoś MZ, Verma N, Cremer D, Kraka E (2023) LModeA2023. Computational and Theoretical Chemistry Group (CATCO), Southern Methodist University, Dallas, TX, USA

[66] Keith TA (2019) AIMAll Version 19.10.12. In: Todd A, Keith TK (eds) Gristmill Software, Overland Park KS, USA. (aim.tkgristmill.com)

[67] Bader RFW (1990) Atoms in molecules: a quantum theory. Oxford University Press, Oxford

[68] Popelier PL (2000) Atoms in molecules: an introduction. Prentice Hall, Essex

[69] Bader RFW (1998) Atoms in molecules. Chem Rev 1:64

[70] Cremer D, Kraka E (1984) Chemical bonds without bonding electron density? does the difference electron-density analysis suffice for a description of the chemical bond? Angew Chem Int Ed 23:627–628 10.1002/anie.198406271

[71] Cremer D, Kraka E (1984) A description of the chemical bond in terms of local properties of electron density and energy. Croatica Chem Acta 57:1259–1281

[72] Cremer D, Wu A, Larsson JA, Kraka E (2000) Some thoughts about bond energies, bond lengths, and force constants. J Mol Model 6:396–412 10.1007/PL00010739

[73] Kraka E, Cremer D (2012) Weaker bonds with shorter bond lengths. Rev Proc Quim 39–42

[74] Setiawan D, Kraka E, Cremer D (2015) Hidden bond anomalies: the peculiar case of the fluorinated amine chalcogenides. J Phys Chem A 119:9541–9556 26280987 10.1021/acs.jpca.5b05157

[75] Kraka E, Setiawan D, Cremer D (2015) Re-evaluation of the bond length-bond strength rule: the stronger bond is not always the shorter bond. J Comp Chem 37:130–142 26515027 10.1002/jcc.24207

[76] Kaupp M, Metz B, Stoll H (2000) Breakdown of bond length-bond strength correlation: a case study. Angew Chem Int Ed 39:4607–4609 10.1002/1521-3773(20001215)39:24<4607::AID-ANIE4607>3.0.CO;2-L 11169686

[77] Kaupp M, Riedel S (2014) On the lack of correlation between bond lengths, dissociation energies, and force constants: the fluorine–substituted ethane homologues. Inorg Chim Acta 357:1865–1872 10.1016/j.ica.2003.11.019

[78] Lindquist BA, Dunning TH Jr (2013) Bonding in FSSF: breakdown in bond length-strength correlations and implications for sf dimerization. J Phys Chem Lett 4:3139–3143 10.1021/jz401578h

[79] Jones LH (1963) Nature of bonding in metal cyanide complexes as related to intensity and frequency of infrared absorption spectra. Inorg Chem 2:777–780 10.1021/ic50008a027

[80] Štellerová D, Lukeš V, Breza M (2023) How does pseudo-Jahn-Teller effect induce the photoprotective potential of curcumin? Molecules 28 10.3390/molecules28072946 PMC10096455 37049707

[81] Hakimi M, Rezaei H, Moeini K, Mardani Z, Eigner V, Dušek M (2020) Formation of a copper-copper bond in coordination of a cyclotriphosphazene ligand toward Cu(II): structural, spectral and docking studies. J Mol Struct 1207:127804

[82] Ito D, Nakao Y, Ishizaki M, Kurihara M, Ando H (2022) Effect of static Jahn–Teller distortion on the Li transport in a copper hexacyanoferrate framework. J Phys Chem A 126:6814–6825 36135963 10.1021/acs.jpca.2c02398

[83] Sigmund LM, Maier R, Greb L (2022) The inversion of tetrahedral p-block element compounds: general trends and the relation to the second-order Jahn-Teller effect. Chem Sci 13:510–521 35126983 10.1039/D1SC05395G PMC8729809

