# Supplementary material for: Exploring Jahn-Teller distortions: a local vibrational mode perspective
Source: J Mol Model. 2024 Mar 13;30(4):102. doi: 10.1007/s00894-024-05882-8 (PMC11315727; doi:10.1007/s00894-024-05882-8)
Supplement: Supplementary file 1 — (pdf 129 KB) [file 894_2024_5882_MOESM1_ESM.pdf]

Supplementary Information:
Exploring Jahn-Teller Distortions: A Local Vibrational Mode Perspective

Mateus Quintano[1], Renaldo T. Moura Jr.[1,2], Elfi Kraka[1*]

[1]Computational and Theoretical Chemistry Group (CATCO), Department of Chemistry, Southern Methodist University, 3215 Daniel Ave, Dallas, TX, 75275-0314, USA.

[2]Department of Chemistry and Physics, Center of Agrarian Sciences, Federal University of Paraiba, Areia, PB, 58397-000, Brazil.

*Corresponding author(s). E-mail(s): ekraka@gmail.com;

Contents

Cartesian Coordinates of the Optimized Geometries **2**

Atomic Charges from the QTAIM Analysis **4**

Cartesian Coordinates of the Optimized Geometries

Cartesian coordinates of the equilibrium structures of test examples **1–4** obtained by unconstrained geometry optimization at the PBE0/Def2-TZVP level of theory [1, 2] using the Gaussian 16 quantum chemistry program [3].

$[Cr(OH_2)_6]^{3+}(T_h)$

Cr	0.00000000	0.00000000	0.00000000
O	0.00000000	0.00000000	1.99711700
O	0.00000000	1.99711700	0.00000000
O	-1.99711700	0.00000000	0.00000000
O	0.00000000	-1.99711700	0.00000000
O	1.99711700	0.00000000	0.00000000
O	0.00000000	0.00000000	-1.99711700
H	0.00000000	-0.78675400	-2.56927000
H	0.00000000	0.78675400	-2.56927000
H	-0.78675400	2.56927000	0.00000000
H	0.78675400	2.56927000	0.00000000
H	0.78675400	-2.56927000	0.00000000
H	-0.78675400	-2.56927000	0.00000000
H	2.56927000	0.00000000	-0.78675400
H	2.56927000	0.00000000	0.78675400
H	0.00000000	0.78675400	2.56927000
H	0.00000000	-0.78675400	2.56927000
H	-2.56927000	0.00000000	-0.78675400
H	-2.56927000	0.00000000	0.78675400

$[Cr(OH_2)_6]^{2+}(D_{2h})$

Cr	0.00000000	0.00000000	0.00000000
O	0.00000000	2.09095700	0.00000000
O	0.00000000	0.00000000	2.08681700
O	2.37403400	0.00000000	0.00000000
O	0.00000000	0.00000000	-2.08681700
O	-2.37403400	0.00000000	0.00000000
O	0.00000000	-2.09095700	0.00000000
H	0.00000000	-2.66073900	-0.77805600
H	0.00000000	-2.66073900	0.77805600
H	0.78107600	0.00000000	2.65344400
H	-0.78107600	0.00000000	2.65344400
H	-0.78107600	0.00000000	-2.65344400
H	0.78107600	0.00000000	-2.65344400
H	-2.95846100	-0.76719200	0.00000000
H	-2.95846100	0.76719200	0.00000000
H	0.00000000	2.66073900	0.77805600
H	0.00000000	2.66073900	-0.77805600
H	2.95846100	-0.76719200	0.00000000

H	2.95846100	0.76719200	0.00000000

$[Fe(CN)_6]^{4-} (O_h)$

Fe	0.00000000	0.00000000	0.00000000
C	0.00000000	0.00000000	1.99199700
C	0.00000000	1.99199700	0.00000000
C	-1.99199700	0.00000000	0.00000000
C	0.00000000	-1.99199700	0.00000000
C	1.99199700	0.00000000	0.00000000
C	0.00000000	0.00000000	-1.99199700
N	0.00000000	0.00000000	3.16576000
N	0.00000000	3.16576000	0.00000000
N	-3.16576000	0.00000000	0.00000000
N	0.00000000	-3.16576000	0.00000000
N	3.16576000	0.00000000	0.00000000
N	0.00000000	0.00000000	-3.16576000

$[Fe(CN)_6]^{3-} (D_{4h})$

Fe	0.00000000	0.00000000	0.00000000
C	0.00000000	1.97611300	0.00000000
C	-1.97611300	0.00000000	0.00000000
C	0.00000000	0.00000000	1.96733800
C	1.97611300	0.00000000	0.00000000
C	0.00000000	0.00000000	-1.96733800
C	0.00000000	-1.97611300	0.00000000
N	0.00000000	3.13899200	0.00000000
N	-3.13899200	0.00000000	0.00000000
N	0.00000000	0.00000000	3.13140200
N	3.13899200	0.00000000	0.00000000
N	0.00000000	0.00000000	-3.13140200
N	0.00000000	-3.13899200	0.00000000

Atomic Charges from the QTAIM Analysis

Atomic charges in atomic units from the QTAIM analysis [4–6] of test examples **1–4** and the isolated ligands using the AIMAll program [7].

$[Cr(OH_2)_6]^{3+}(T_h)$

```
Cr1 +2.051146
O2 -1.224734
O3 -1.224781
O4 -1.224757
O5 -1.224743
O6 -1.224714
O7 -1.224728
H8 +0.691485
H9 +0.691482
H10 +0.691475
H11 +0.691488
H12 +0.691489
H13 +0.691476
H14 +0.691485
H15 +0.691482
H16 +0.691485
H17 +0.691484
H18 +0.691486
H19 +0.691482
    +3.000486
```

$[Cr(OH_2)_6]^{2+}(D_{2h})$

```
Cr1 +1.613966
O2 -1.231121
O3 -1.235113
O4 -1.229514
O5 -1.235136
O6 -1.229530
O7 -1.231093
H8 +0.654426
H9 +0.654427
H10 +0.657581
H11 +0.657584
H12 +0.657584
H13 +0.657581
H14 +0.632330
H15 +0.632328
H16 +0.654428
H17 +0.654427
```

```
H18 +0.632340
H19 +0.632337
    +1.999832
```

$H_2O(C_{2v})$

```
O1 -1.149847
H3 +0.574922
H2 +0.574922
    -0.000004
```

$[Fe(CN)_6]^{4-}(O_h)$

```
Fe1 +1.031385
C2 +0.815039
C3 +0.815074
C4 +0.814991
C5 +0.814976
C6 +0.814947
C7 +0.815049
N8 -1.653613
N9 -1.653608
N10 -1.653611
N11 -1.653615
N12 -1.653614
N13 -1.653612
    -4.000213
```

$[Fe(CN)_6]^{3-}(D_{4h})$

```
Fe1 +1.233599
C2 +0.816527
C3 +0.816416
C4 +0.824986
C5 +0.816432
C6 +0.824977
C7 +0.816441
N8 -1.514338
N9 -1.514339
N10 -1.545868
N11 -1.514344
N12 -1.545868
N13 -1.514345
    -2.999724
```

$CN^-(C_{\infty v})$

```
N1 -1.608013
C2 +0.608012
    -1.000001
```

References

[1] Adamo, C., Barone, V.: Toward reliable density functional methods without adjustable parameters: The PBE0 model. J. Chem. Phys. **110**, 6158–6170 (1999)

[2] Weigend, F., Ahlrichs, R.: Balanced basis sets of split valence, triple zeta valence and quadruple zeta valence quality for H to Rn: Design and assessment of accuracy. Phys. Chem. Chem. Phys. **7**, 3297–3305 (2005)

[3] Frisch, M.J., Trucks, G.W., Schlegel, H.B., Scuseria, G.E., Robb, M.A., Cheeseman, J.R., Scalmani, G., Barone, V., Petersson, G.A., Nakatsuji, H., Li, X., Caricato, M., Marenich, A.V., Bloino, J., Janesko, B.G., Gomperts, R., Mennucci, B., Hratchian, H.P., Ortiz, J.V., Izmaylov, A.F., Sonnenberg, J.L., Williams-Young, D., Ding, F., Lipparini, F., Egidi, F., Goings, J., Peng, B., Petrone, A., Henderson, T., Ranasinghe, D., Zakrzewski, V.G., Gao, J., Rega, N., Zheng, G., Liang, W., Hada, M., Ehara, M., Toyota, K., Fukuda, R., Hasegawa, J., Ishida, M., Nakajima, T., Honda, Y., Kitao, O., Nakai, H., Vreven, T., Throssell, K., Montgomery, J.A. Jr., Peralta, J.E., Ogliaro, F., Bearpark, M.J., Heyd, J.J., Brothers, E.N., Kudin, K.N., Staroverov, V.N., Keith, T.A., Kobayashi, R., Normand, J., Raghavachari, K., Rendell, A.P., Burant, J.C., Iyengar, S.S., Tomasi, J., Cossi, M., Millam, J.M., Klene, M., Adamo, C., Cammi, R., Ochterski, J.W., Martin, R.L., Morokuma, K., Farkas, O., Foresman, J.B., Fox, D.J.: Gaussian 16 Revision C.01. Gaussian Inc. Wallingford CT (2016)

[4] Bader, R.F.W.: Atoms in Molecules: A Quantum Theory. Oxford University Press, Oxford (1990)

[5] Popelier, P.L.: Atoms in Molecules: An Introduction. Prentice Hall, Essex (2000)

[6] Bader, R.F.W.: Atoms in Molecules. Chem. Rev. **1**, 64 (1998)

[7] Keith, T.A.: AIMAll Version 19.10.12. Todd A. Keith, TK Gristmill Software, Overland Park KS, USA. (aim.tkgristmill.com) (2019)
